# A Radiomics Approach to Identify Immunologically Active Tumor in Patients with Head and Neck Squamous Cell Carcinomas

**DOI:** 10.3390/cancers15225369

**Published:** 2023-11-11

**Authors:** Tan Mai Nguyen, Chloé Bertolus, Paul Giraud, Anita Burgun, Pierre Saintigny, Jean-Emmanuel Bibault, Jean-Philippe Foy

**Affiliations:** 1 Sorbonne Université, Department of Maxillo-Facial Surgery, Hôpital Pitié-Salpêtrière, Assistance Publique des Hôpitaux de Paris, 75013 Paris, France; tanmai.nguyen@aphp.fr (T.M.N.); chloe.bertolus@aphp.fr (C.B.); 2 Univ Lyon, Université Claude Bernard Lyon 1, INSERM 1052, CNRS 5286, Centre Léon Bérard, Centre de Recherche en Cancérologie de Lyon, 69008 Lyon, France; pierre.saintigny@lyon.unicancer.fr; 3 INSERM, UMR S1138, Cordeliers Research Center, Université Paris Cité, 75005 Paris, France; p-giraud@outlook.com (P.G.); anita.burgun@aphp.fr (A.B.); jean-emmanuel.bibault@aphp.fr (J.-E.B.); 4 Sorbonne Université, Department of Radiation Oncology, Hôpital Pitié-Salpêtrière, Assistance Publique des Hôpitaux de Paris, 75013 Paris, France; 5 Department of Medical Oncology, Centre Léon Bérard, 69008 Lyon, France; 6 Department of Radiation Oncology, Hôpital Européen Georges-Pompidou, Université Paris Cité, 75015 Paris, France; 7 Sorbonne Université, INSERM UMRS 938, Centre de Recherche de Saint Antoine, Team Cancer Biology and Therapeutics, 75011 Paris, France

**Keywords:** head and neck squamous cell carcinoma, radiomic, hot phenotype, biomarker

## Abstract

**Simple Summary:**

We recently established a biological classification of head and neck cancers into hot and cold tumors, which were associated with different responses to immunotherapy (a type of cancer treatment helping the immune system fight cancer). Because this classification requires a tumor biopsy, the development of a non-invasive approach, based on imaging data, would be relevant to determine this hot/cold tumor status without performing an invasive biopsy. Thus, our goal was to determine whether imaging data from computed tomography (CT) scans can distinguish hot and cold head and neck squamous cell carcinomas (HNSCCs). Using biological and clinical imaging data from two independent cohorts, we established a computational model to determine the hot/cold status from the CT scan. This non-invasive approach, based on a “virtual” biopsy, could help for the identification and monitoring of patients with HNSCC who may benefit from immunotherapy.

**Abstract:**

Background: We recently developed a gene-expression-based HOT score to identify the hot/cold phenotype of head and neck squamous cell carcinomas (HNSCCs), which is associated with the response to immunotherapy. Our goal was to determine whether radiomic profiling from computed tomography (CT) scans can distinguish hot and cold HNSCC. Method: We included 113 patients from The Cancer Genome Atlas (TCGA) and 20 patients from the Groupe Hospitalier Pitié-Salpêtrière (GHPS) with HNSCC, all with available pre-treatment CT scans. The hot/cold phenotype was computed for all patients using the HOT score. The IBEX software (version 4.11.9, accessed on 30 march 2020) was used to extract radiomic features from the delineated tumor region in both datasets, and the intraclass correlation coefficient (ICC) was computed to select robust features. Machine learning classifier models were trained and tested in the TCGA dataset and validated using the area under the receiver operator characteristic curve (AUC) in the GHPS cohort. Results: A total of 144 radiomic features with an ICC >0.9 was selected. An XGBoost model including these selected features showed the best performance prediction of the hot/cold phenotype with AUC = 0.86 in the GHPS validation dataset. Conclusions and Relevance: We identified a relevant radiomic model to capture the overall hot/cold phenotype of HNSCC. This non-invasive approach could help with the identification of patients with HNSCC who may benefit from immunotherapy.

## 1. Introduction

Head and neck squamous cell carcinomas (HNSCCs) are common epithelial tumors arising from the mucosa of the oral cavity, oropharynx, nasopharynx, larynx, or hypopharynx. They are ranked as the 7th most common cancers worldwide with over 800,000 patients diagnosed per year globally, and they are responsible for over 400,000 deaths [1]. HNSCC is characterized by a high inter- and intra-tumoral heterogeneity, which may significantly impact treatment response.

Recent clinical trials evaluating immunotherapies, notably PD-1/PD-L1 inhibitors, represent an unprecedented advance in the management of HNSCC [2,3]. Beyond the identification of a specific biomarker associated with the response to a given immune checkpoint inhibitor (ICI), the upcoming era of new immunotherapy strategies combining different ICIs with co-stimulatory agonists or therapeutic vaccines, as well as with cytotoxic drugs, has recently led to the concept of targeting hot versus cold tumors [4,5].

Briefly, compared to cold tumors, hot tumors have been characterized by an overall activated immune microenvironment with high infiltrating immune cells and are more likely to respond to immunomodulatory strategies. Although the concept of hot versus cold tumors is questionable regarding the known complexity of the immune microenvironment [6], it is of particular relevance in clinical research for the identification of patients more likely to benefit from immunotherapies, including in neoadjuvant settings. In this context, we recently developed an HOT score based on the expression of 27 genes in order to identify hot head and neck tumors [7]. We showed that hot tumors, as defined by a positive HOT score, were characterized by a high TCD8 (T-cell) infiltrate as well as an activation of the interferon-gamma (IFN-γ) response. Interestingly, in two independent cohorts of patients with HNSCC or non-small-cell lung cancers (NSCLCs) treated with immunotherapies, we also showed that patients with hot tumors had a better overall survival as well as progression-free survival compared to cold tumors.

Medical imaging is routinely used for cancer patients and is promising in the development of non-invasive biomarkers for the molecular characterization of a tumor phenotype [8]. Computational medical imaging, known as radiomics, is an emerging field first defined in 2012 as the high-throughput extraction of large amounts of image features from radiographic images [9,10]. Radiomics provides fast, low-cost, and non-invasive instruments through the generation of image-driven biomarkers directly extracted from standard-of-care medical images. Over the past few years, we have observed an increasing trend of publications on radiomic approaches in oncology [11], especially for predicting diagnosis [12,13,14] and treatment response [15,16,17,18,19], as well as tumor molecular profiles [20,21]. In particular, in patients with HNSCC, different radiomic features extracted from computed tomography (CT) imaging, which is routinely performed in this setting, were significantly associated with clinical (local control, tumor failure, lymph node metastasis, extranodal extension, overall survival, human papillomavirus (HPV) status) and histological (differentiation grade, perineural and lymphovascular invasion) parameters as well as molecular features, especially some specific somatic mutations, in patients with head and neck cancer [22,23,24,25,26,27,28,29,30,31,32,33,34,35,36,37,38,39].

Because some radiomic features were also associated with the tumor immune microenvironment of HNSCC, such as the T-cell infiltration [40], we hypothesized that radiomic analysis can predict the immune tumor hot phenotype of HNSCC. Using radiomic features extracted from pretreatment CT images of HNSCC, we developed a radiomic model for tumor classification according to the hot/cold phenotype.

## 2. Methods

### 2.1. Datasets (Table 1)

#### 2.1.1. Training and Testing Datasets

The “TCGA-HNSCC” dataset includes molecular and imaging data of patients with HNSCC from The Cancer Genome Atlas database. Gene expression profiles as well as clinical data were downloaded using the TCGA2STAT R package [41] as well as from the cBioportal database (https://www.cbioportal.org/study/summary?id=hnsc_tcga (accessed on 3 March 2020) [42,43]. DICOM files of corresponding pre-treatment CT scans were downloaded from The Cancer Imaging Archive (TCIA) (https://www.cancerimagingarchive.net/nbia-search/?CollectionCriteria=TCGA-HNSC (accessed on 3 March 2020) (N = 188).

#### 2.1.2. External Validating Dataset

The “GHPS” dataset included 20 patients treated in the department of maxillo-facial surgery at the Groupe Hospitalier la Pitié-Salpétrière (GHPS), with available gene expression profiles as well as corresponding pre-treatment CT scans (DICOM images). For each patient, targeted gene expression profiles were previously generated in order to define the hot/cold tumor phenotype based on the HOT score as previously defined [7,44]. We retrospectively reviewed corresponding contrast-enhanced pre-treatment CT scans and data were de-identified. For both datasets, inclusion criteria were patients suffering from histologically confirmed HNSCC with available pretreatment contrast-enhanced CT scans. Only patients with CT scans of the proper quality were included (contrast-enhanced CT, soft or standard convolution kernel, slice thickness ≤ 5 mm, no artifacts (dental) in the region of interest, visualization of the tumor).

In all samples from the training and validating datasets, we computed the HOT score using the gene set variation analysis (GSVA) [45], as previously defined, in order to identify the hot/cold tumor. Tumors with a score ≥ 0 and <0 were classified as hot and cold, respectively.

Tumor characteristics are detailed for each dataset in Table 1.

**Table 1:** Patient characteristics of the two cohorts. NA: not available.

	TCGA-HNSCC (N = 113)	GHPS-COSMOS (N = 20)
	N (%)	N (%)
Age (mean) at diagnosis	59.69	62.15
Gender		
Female	27 (23.9%)	8 (40.0%)
Male	86 (76.1%)	12 (60.0%)
Anatomic Site		
Oral cavity	68 (60.2%)	20 (100%)
Larynx	31 (27.4%)	0
Oropharynx	13 (11.5%)	0
Hypopharynx	1 (0.9%)	0
Alcohol		
Yes	28 (24.8%)	8 (40.0%)
No	85 (75.2%)	10 (50.0%)
NA	0	2 (10.0%)
Smoking		
Current	45 (39.8%)	10 (50.0%)
Former	46 (40.7%)	2 (10.0%)
No	21 (18.6%)	8 (40.0%)
NA	1 (0.9%)	0
Pathological T stage		
T1	6 (5.3%)	1 (5.0%)
T2	23 (20.3%)	5 (25.0%)
T3	23 (30.3%)	3 (10.0%)
T4	41 (36.3%)	11 (55.0%)
Tx	13 (38.0%)	0
NA	5 (11.5%)	0
N stage (%)		
N0	47 (41.6%)	10 (50%)
N1	13 (11.5%)	2 (10%)
N2	32 (28.3%)	4 (20%)
N3	1 (0.9%)	4 (20%)
Nx	15 (13.3%)	0
NA	5 (4.4%)	0
M stage (%)		
M0	109 (96.5%)	20 (100%)
M1	1 (0.9%)	0
Mx	3 (2.6%)	0
Phenotype (%)		
HOT	48 (42.5%)	9 (45%)
COLD	65 (57.5%)	11 (55%)

### 2.2. Radiomics Workflow (Figure 1)

#### 2.2.1. Tumor Volume Segmentation

We manually outlined the regions of interest (ROIs, defined as the gross tumor volume) using the “Imaging Biomarker Explorer” IBEX radiomics software [46,47] on each axial slice. During this process of segmentation, all artifacts and other tissues around the tumor as well as airways were avoided in order to include only tumor tissues in the ROIs. All the ROIs were segmented by a head and neck specialist (T.-M.N.). In the TCGA dataset, a second delineation was performed on 17 randomly selected CT scans by a radiation oncologist (J.-E.B.). This repeated manual segmentation was performed to assess the reproducibility and stability of radiomic features in light of the variation in manual selection. The ROIs from the two independent specialists were downloaded into the IBEX software and compared. Divergences in the two segmentations for each tumor volume were resolved through discussion with another head and neck expert (J.-P.F.).

#### 2.2.2. Quantitative Image Feature Extraction

The IBEX software automatically generates tumor radiomic features in six principal categories: shape, intensity histogram, gray level co-occurrence matrix (GLCOM) 25 (computed from all 2-D image slices) and 3 (computed from all 3-D image matrices), neighborhood gray-tone difference matrix (NGTDM) 3 and 25, gray level run-length matrix (GLRLM) 25, and histogram of oriented gradients (Supplementary Table S1). A total of 1767 quantitative image features were extracted from the segmented region of interest of each gross tumor volume, without image preprocessing filters.

#### 2.2.3. Feature Selection

Extracted radiomic feature scaling was performed through normalization in a linear manner within the range [0:1]. To assess the robustness of the radiomic features from the ROIs, we computed the intraclass correlation coefficient (ICC) for each radiomic feature [48]. Thus, the ICC can be used when quantitative measurements are performed on units organized into groups [49]. It ranges from 0 to 1, indicating null and perfect reproducibility, respectively. We used the “ICC” R package (version 3.4.2) to calculate this coefficient (https://cran.r-project.org/web/packages/ICC/ICC.pdf (accessed on 20 May 2020). Only features with an ICC greater than or equal to 0.90 were selected.

**Figure 1:**
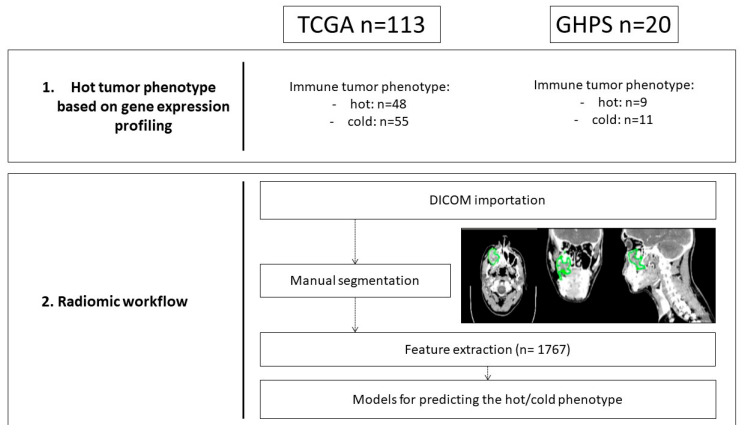
Radiomic workflow.

### 2.3. Radiomic Model (Figure 2)

Selected features were used as predictor inputs for constructing XGBoost models, which is a decision-tree-based ensemble machine learning algorithm using a gradient-boosting framework for regression and classification problems. This model uses a large number of “weak learners,” which are relatively simple classifiers, each trained using only part of the data. The estimate produced by each weak learner is then aggregated into an overall decision using mechanisms such as voting. In this manner, a large number of weak learners are combined to produce one strong learner. Thus, a sequential training process creates a cascade of weak learners, such that in each subsequent step, weak learners focus on the data that were most difficult to classify in the previous step. To improve our model, we performed hyperparameter tuning using a grid search. Moreover, to limit overfitting and increase the generalizability of our models, we constructed our model based on training, validation, and independent testing in separate datasets [50]: the TCGA dataset was randomly divided into the training dataset (80%) and the testing dataset (20%), while the GHPS cohort was an independent validating dataset. The characteristics of the datasets are available in Supplementary Table S2. Finally, to minimize the true error estimate bias, each classifier model was trained using three-fold cross-validation (3-fold CV) on the training set [51]. We estimated the contribution of each variable to the model using the varImp function from the R caret package.

In order to assess the predictive performance of our model, we computed different well-established metrics from the literature. Notably, we measured the area under the receive operator characteristic (ROC) curve (AUC) using the pROC package in R. We also computed accuracy, recall, precision, F-score, and area under the precision–recall curve using the precrec package in R (https://cran.r-project.org/web/packages/precrec/vignettes/introduction.html (accessed on 10 September 2020). AUC and accuracy are two of the most commonly used classification metrics in machine learning. AUC is commonly recognized as the best indicator of model performance, especially due to the relationship between true positive rate and false positive rate [52], except in the case of class imbalance. In this context and because the hot and cold classes are well balanced, we used AUC to select the best model for the classification of the hot/cold phenotype. A brief description of each performance metric is provided in Supplementary Table S3.

All the machine learning algorithms were conducted using the R caret package [50]. All the analyses were completed using the R software (version R 4.0.1) [51]. The code is available at https://github.com/TMNgn/Master2IBM.git (accessed on 3 March 2020).

**Figure 2:**
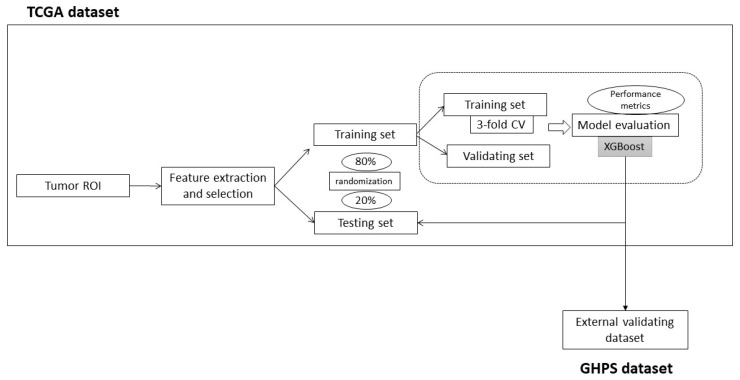
Model construction.

## 3. Results

### 3.1. Identification of the Hot Phenotype and Radiomic Feature Selection

A total of 113 and 20 patients suffering from HNSCC were included from the TCGA-HNSCC and the GHPS cohorts, respectively. The hot/cold phenotype was previously computed using the HOT score in each sample. We identified 48/113 and 9/20 hot tumors from the TCGA and GHPS cohorts, respectively.

We initially extracted 1767 radiomic features from the segmented tumor regions of the pretreatment CT images from the TCGA-HNSCC cohort. The ICC was computed from the tumor ROI delineation by two independent investigators (T.-M.N. and J.-E.B.) in 17 randomly selected patients. A total of 144/1767 features with ICC ≥ 0.90 were selected for further analysis (Supplementary Table S3).

### 3.2. Predictive Radiomic Model of the Hot Phenotype

Using the 144 selected radiomic features, we trained an XGBoost model on the training set of the TCGA cohort and tuned the hyperparameters.

**Figure 3:**
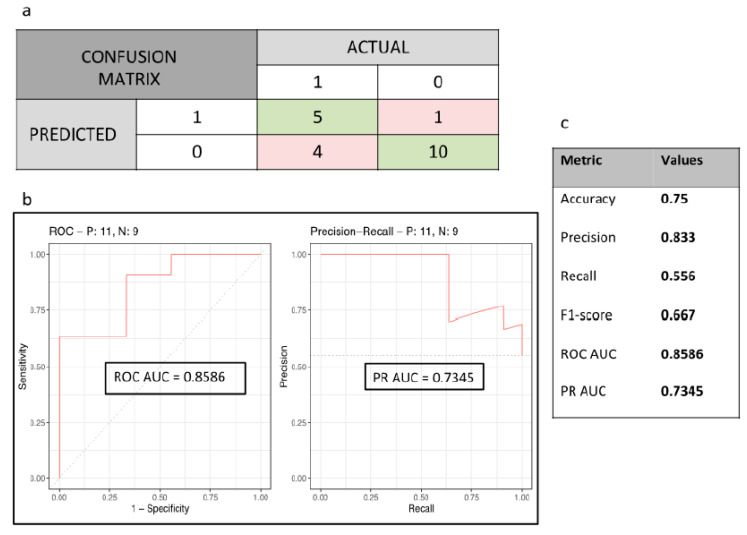
Best XGBoost prediction model of the HOT–COLD phenotype in the GHPS cohort. (**a**) Confusion matrix. (**b**) ROC curve and precision–recall curve. (**c**) Performance metrics values.

Our best XGBoost model after hyperparameter tuning (booster = gbtree, nrounds = 4900, eta = 0.01, max_depth = 6, gamma = 0.5, colsample_bytree = 0.6, min_child_weight = 1, subsample = 1) on the independent validating cohort (GHPS) predicted the hot/cold phenotype with the following performance metrics: AUC = 0.859, PR-AUC = 0.734, accuracy = 0.750, precision = 0.833, recall = 0.556, and F1-score = 0.667 (Figure 3). The confusion matrix and the model performance metrics are summarized in Figure 3a,c. The top five contributive radiomic features using the varImp function from the R caret package were F10.Shape Voxel Size, F1.Gradient Orient Histogram 0.975 Quantile, F1.Gradient Orient Histogram 85 Percentile, F1.Gradient Orient Histogram 95 Percentile Area, and F2. Gray Level Co-occurence Matrix 25270.7 Auto Correlation.

The min–max values of the performance metrics of all the classification methods trained with the selected features are reported in Supplementary Table S4.

## 4. Discussion

Medical imaging is routinely used for cancer patients and is promising in the development of non-invasive biomarkers for the molecular characterization of a tumor phenotype [53]. While most radiomic studies in patients with HNSCC focused on diagnosis and prognosis prediction [16,23,24,25,27,54,55], few publications only showed the association between radiomic features and the molecular characteristics of head and neck cancers, especially involving the immune tumor microenvironment [40,56,57,58,59]. Because we previously demonstrated the association between a hot/cold tumor phenotype based on gene expression and the association with the response to anti-PD1/PD-L1 in patients with HNSCC, we assessed whether some radiomic features extracted from the tumor ROI could help with the prediction of this molecular phenotype. Based on initial CT scans, we built and tested a radiomic XGBoost model, including 144 robust features, to predict our hot/cold tumor phenotype. Notably, we observed the good performance of our model for the prediction of the hot/cold phenotype, with AUC = 0.86. Besides the performance metrics, this study showed the relevance of a non-invasive imaging radiomics-based approach to capture molecular tumor characteristics in HNSCC.

The association between radiomics and cancer biology, especially regarding the tumor immune microenvironment, has been recently investigated in some cancer types, especially in lung and breast cancers [60,61,62,63,64,65,66,67]. Some studies have focused on HNSCC [60,68], with most of them investigating the relevance of radiomics to capture histological features related to HPV status [25] and immune cell infiltration [40]. Indeed, different radiomic signatures were identified to assess tumor T-CD8 cell infiltration [69] as well as PD-L1 expression or other immune checkpoint molecules [58,59,70,71], which were associated with the response to PD1/PD-L1 inhibitors. Interestingly, some radiomic features were also associated with the histological classification between immune-inflamed and immune-desert tumors [59]. However, the inter- and intra-observer variability in the pathological assessment of immune features in cancer samples, especially PDL1 expression [72], may limit the extrapolability of radiomic signatures for capturing histological immune features, suggesting the need to identify radiomic surrogates of the molecular biomarkers of the immune infiltrate. In line with previous studies showing the relevance of radiomic models to identify gene-expression- and methylome-based subtypes [56], with distinct immune molecular features [40], we built a radiomic model to classify hot and cold tumors, as defined by our score based on the expression of 27 genes. Thus, our study confirms, in patients with HNSCC, the relevance of a non-invasive imaging approach to identify different immune subtypes, as previously performed in other cancer types [63,64,73,74,75].

Our model was based on an XGBoost method, which is a relevant machine learning algorithm for problem classification [76]. This algorithm has recently been dominating applied machine learning and Kaggle competitions for structured or tabular data, and it has proven its relevance in building radiomic classification models for different cancer types [28,77,78,79,80]. This model includes a large number of hyperparameters that are divided into general parameters (booster), booster parameters (eta, min_child_weight, max_depth, max_leaf_nodes, gamma, max_ delta_step, subsample, etc.), and learning task parameters (objective, eval_metric, and seed). Thus, we performed exhaustive parameter tuning in this model in order to improve the predictive performance of our model. Hyperparameter tuning as well as the cross-validation method and feature selection were used to limit overfitting, as previously recommended [81], with very good predictive performance (AUC = 0.86) in the external validation dataset.

We observed that shape and textural radiomic features were the most relevant quantitative image features that contributed to the predictive performance of our model. This observation is consistent with recent reports of radiomics and biology in HNSCC, showing that textural features were associated with tumor heterogeneity and were commonly used in radiomic model biological prediction [68,71]. In particular, image-derived textural diversity has been associated with increased immune cell infiltration, causing tumor imaging heterogeneity [65,82]. Although the relevance of radiomic features to identify molecular immune features is questionable regarding the known complexity of the immune microenvironment, the very good predictive performances of our model may also be related to a relatively simple binary classification with major biological differences between hot and cold tumors [7], which goes beyond the expression of a single gene or protein biomarker.

The principal limitation of our study is the relatively small sample size (<150 patients). However, the TCGA dataset was split into a training and a testing dataset to build a model that we validated in an independent dataset, strengthening confidence in the generalizability of our model. Indeed, the inter-scanner variability in CT image radiomic studies especially related to scanner manufacturers [83] may limit the extrapolability of previously published radiomic models using training and validation from a single institutional dataset. Because the TCGA and GHPS datasets are two independent datasets with different imaging parameters, we believe that our radiomic model is robust and could be extrapolated in other centers to predict this hot/cold molecular phenotype. With the goal of the further clinical application of our model in routine practice to predict clinical outcomes of patients treated with immunotherapies, larger institutional cohorts are needed to validate our model, with the possibility of “face to face” verification of the clinical characteristics and the natural evolution of the disease in every single case. Moreover, this model was constructed from manually delineated ROIs. Although the variability in manual segmentation may cause intra- and inter-reader variability [84], a second delineation was performed by an independent investigator in order to select stable features by computing the ICC. Semi-automated approaches can help reduce this variability but are still reliant on human input [85]. Although automatic segmentation techniques are objective, they may generate errors, especially when images have artifacts and noise and when lesions of interest are highly heterogeneous.

## 5. Conclusions

In conclusion, we identified a robust radiomic model using an XGBoost classifier to predict gene-expression-based hot and cold phenotypes, which were associated with the clinical benefit of PD1/PD-L1 inhibitors. Overall, this non-invasive-imaging-based approach, called a “virtual biopsy”, could help with the selection of patients for treatment with immunotherapies in different clinical settings: (i) before surgery in order to refine neoadjuvant immunomodulatory strategies based on the hot/cold phenotype and (ii) in patients with recurrent and/or metastatic disease in order to avoid repeated invasive biopsies and/or technically challenging biopsies. Moreover, compared to molecular analysis, the cost-effectiveness of radiomics is a crucial point when considering the incorporation of radiomic signatures/models within future clinical trials for precision medicine.

## Data Availability

Clinical data as well as gene expression profiles are publicly available from The Cancer Genome Atlas and Gene Expression Omnibus (GSE159067).

## Supplementary Materials

The following supporting information can be downloaded at: https://www.mdpi.com/article/10.3390/cancers15225369/s1, Table S1: Radiomics features used in the analysis; Table S2: Subtypes characteristics in the different datasets; Table S3: 144 radiomic features used for the analysis; Table S4: Performance metrics with XG Boost models to predict the hot/cold phenotype.
