# Supplementary material for: A Radiomics Approach to Identify Immunologically Active Tumor in Patients with Head and Neck Squamous Cell Carcinomas
Source: Cancers (Basel). 2023 Nov 11;15(22):5369. doi: 10.3390/cancers15225369 (PMC10670096; doi:10.3390/cancers15225369)
Supplement: Supplementary file 1 [file cancers-15-05369-s001.zip › cancers-2651131-supplementary.pdf]

Supplementary Table S1. Radiomics features used in the analysis

Gray Level Co-occurrence Matrix	Gray Level Run Length Matrix	Intensity Histogram	Neighborhood Gray Tone Difference Matrix	Gradient Orient Histogram	Shape
Auto Correlation	Gray Level Nonuniformity	Energy	Busyness	Inter Quartil Range Kurtosis	Compactness 1
Cluster Prominence	High Gray Level	Entropy	Coarseness	Mean Absolute Deviation	Compactness 2
Cluster Shade	Run Emphasis	Kurtosis	Complexity	Median Absolute Deviation	Convex
Cluster Tendency	Long Run Emphasis	Mean	Contrast	Percentile	Convex Hull Volume
Contrast Correlation	Long Run High Gray Level Emphasis	Median	Texture	Percentile Area	Convex Hull Volume 3D
Difference Entropy	Long Run Low Gray Level Emphasis	Minimum	Strength	Randge	Mass
Dissimilarity	Low Gray Level Run Emphasis	Skewness		Skewness	Max3DDiameter
Energy	Run Length Nonuniformity	Standard			Mean Breadth
Entropy	Run Percentage	Deviation			Number of objects
Homogeneity	Short Run Emphasis	Uniformity			Number of voxel
Homogeneity 2		Variance			Orientation
Information Measure Correlation 1					Roundness
Information					Spherical Disproportion
					Shericity
					Surface Area

Measure Correlation 2	Short Run High Gray Level Emphasis			Surface Area Density
Inverse Difference Moment Norm	Short Run Low Gray Level Emphasis			Volume
Inverse Difference Norm				Voxel Size
Inverse Variance				
Max Probability				
Sum Average				
Sum Entropy				
Sum Variance				
Variance				

Supplementary Table S2. Subtypes characteristics in the different datasets

Class/Phenotype	TCGA dataset %	TCGA - training dataset 80% (90/113)	TCGA-testing 20% (23/113)	GHPS %	SMOTE TCGA dataset
**Hot**	42.5 (48/113)	45.6 (41/90)	30.4 (7/23)	45 (9/20)	48.67 (55/113)
**Cold**	57.5 (65/113)	54.4 (49/90)	69.6 (16/23)	55 (11/20)	51.33 (58/113)

Supplementary Table S3. 144 radiomic features used for the analysis

**Shape**	
1/144 (0.7%)	F10.ShapeVoxelSize
**Intensity Histogram**	
47/144 (32.6%)	F5.IntensityDirectGlobalMedian<br>F5.IntensityDirectLocalEntropyMedian<br>F5.IntensityDirectLocalEntropyMin<br>F5.IntensityDirectLocalRangeMedian<br>F5.IntensityDirectLocalRangeMin<br>F5.IntensityDirectLocalStdMedian<br>F5.IntensityDirectLocalStdMin<br>F5.IntensityDirect45Percentile<br>F5.IntensityDirect50Percentile<br>F5.IntensityDirect55Percentile<br>F5.IntensityDirect60Percentile<br>F5.IntensityDirect65Percentile<br>F5.IntensityDirect70Percentile<br>F5.IntensityDirect75Percentile<br>F5.IntensityDirect80Percentile<br>F5.IntensityDirect85Percentile<br>F5.IntensityDirect90Percentile<br>F5.IntensityDirect95Percentile<br>F5.IntensityDirect..0.5Quantile<br>F5.IntensityDirect.0.75Quantile<br>F5.IntensityDirect0.975Quantile<br>F6.IntensityHistogram45Percentile<br>F6.IntensityHistogram50Percentile<br>F6.IntensityHistogram55Percentile<br>F6.IntensityHistogram60Percentile<br>F6.IntensityHistogram65Percentile<br>F6.IntensityHistogram70Percentile<br>F6.IntensityHistogram75Percentile<br>F6.IntensityHistogram80Percentile<br>F6.IntensityHistogram85Percentile<br>F6.IntensityHistogram90Percentile<br>F6.IntensityHistogram95Percentile<br>F6.IntensityHistogram45PercentileArea<br>F6.IntensityHistogram50PercentileArea<br>F6.IntensityHistogram55PercentileArea<br>F6.IntensityHistogram60PercentileArea<br>F6.IntensityHistogram65PercentileArea

	F6.IntensityHistogram70PercentileArea
	F6.IntensityHistogram75PercentileArea
	F6.IntensityHistogram80PercentileArea
	F6.IntensityHistogram85PercentileArea
	F6.IntensityHistogram90PercentileArea
	F6.IntensityHistogram95PercentileArea
	F6.IntensityHistogram..0.5Quantile
	F6.IntensityHistogram.0.75Quantile
	F6.IntensityHistogram0.975Quantile
	F7.IntensityHistogramGaussFit1GaussMean
**Gray level co-occurrence matrix (GLCOM) 25 and 3**	
63/144 (43.8 %)	F2.GrayLevelCooccurenceMatrix2590.7AutoCorrelation
	F2.GrayLevelCooccurenceMatrix25270.7AutoCorrelation
	F2.GrayLevelCooccurenceMatrix25.333.1InformationMeasureCorr1
	F2.GrayLevelCooccurenceMatrix250.1InformationMeasureCorr1
	F2.GrayLevelCooccurenceMatrix2590.1InformationMeasureCorr1
	F2.GrayLevelCooccurenceMatrix25180.1InformationMeasureCorr1
	F2.GrayLevelCooccurenceMatrix25270.1InformationMeasureCorr1
	F2.GrayLevelCooccurenceMatrix25.333.1InverseVariance
	F2.GrayLevelCooccurenceMatrix250.1InverseVariance
	F2.GrayLevelCooccurenceMatrix2545.1InverseVariance
	F2.GrayLevelCooccurenceMatrix2590.1InverseVariance
	F2.GrayLevelCooccurenceMatrix25135.1InverseVariance
	F2.GrayLevelCooccurenceMatrix25180.1InverseVariance
	F2.GrayLevelCooccurenceMatrix25225.1InverseVariance
	F2.GrayLevelCooccurenceMatrix25270.1InverseVariance
	F2.GrayLevelCooccurenceMatrix25315.1InverseVariance
	F2.GrayLevelCooccurenceMatrix2590.7SumAverage
	F2.GrayLevelCooccurenceMatrix25270.7SumAverage
	F3.GrayLevelCooccurenceMatrix30.7AutoCorrelation
	F3.GrayLevelCooccurenceMatrix39.4AutoCorrelation
	F3.GrayLevelCooccurenceMatrix32.1Homogeneity
	F3.GrayLevelCooccurenceMatrix35.1Homogeneity
	F3.GrayLevelCooccurenceMatrix36.1Homogeneity
	F3.GrayLevelCooccurenceMatrix37.1Homogeneity
	F3.GrayLevelCooccurenceMatrix38.1Homogeneity
	F3.GrayLevelCooccurenceMatrix39.1Homogeneity
	F3.GrayLevelCooccurenceMatrix310.1Homogeneity
	F3.GrayLevelCooccurenceMatrix32.1Homogeneity2
	F3.GrayLevelCooccurenceMatrix35.1Homogeneity2
	F3.GrayLevelCooccurenceMatrix36.1Homogeneity2
	F3.GrayLevelCooccurenceMatrix37.1Homogeneity2

	F3.GrayLevelCooccurenceMatrix38.1Homogeneity2
	F3.GrayLevelCooccurenceMatrix39.1Homogeneity2
	F3.GrayLevelCooccurenceMatrix310.1Homogeneity2
	F3.GrayLevelCooccurenceMatrix30.1InformationMeasureCorr1
	F3.GrayLevelCooccurenceMatrix31.1InformationMeasureCorr1
	F3.GrayLevelCooccurenceMatrix3.333.1InverseVariance
	F3.GrayLevelCooccurenceMatrix30.1InverseVariance
	F3.GrayLevelCooccurenceMatrix31.1InverseVariance
	F3.GrayLevelCooccurenceMatrix32.1InverseVariance
	F3.GrayLevelCooccurenceMatrix33.1InverseVariance
	F3.GrayLevelCooccurenceMatrix34.1InverseVariance
	F3.GrayLevelCooccurenceMatrix38.1InverseVariance
	F3.GrayLevelCooccurenceMatrix30.7SumAverage
	F3.GrayLevelCooccurenceMatrix39.4SumAverage
	F4.GrayLevelRunLengthMatrix25.333LongRunEmphasis
	F4.GrayLevelRunLengthMatrix25...0LongRunEmphasis
	F4.GrayLevelRunLengthMatrix25..90LongRunEmphasis
	F4.GrayLevelRunLengthMatrix25.333LongRunHighGrayLevelEmpha
	F4.GrayLevelRunLengthMatrix25...0LongRunHighGrayLevelEmpha
	F4.GrayLevelRunLengthMatrix25..90LongRunHighGrayLevelEmpha
	F4.GrayLevelRunLengthMatrix25.333LongRunLowGrayLevelEmpha
	F4.GrayLevelRunLengthMatrix25...0LongRunLowGrayLevelEmpha
	F4.GrayLevelRunLengthMatrix25..90LongRunLowGrayLevelEmpha
	F4.GrayLevelRunLengthMatrix25.333RunLengthNonuniformity
	F4.GrayLevelRunLengthMatrix25...0RunLengthNonuniformity
	F4.GrayLevelRunLengthMatrix25..90RunLengthNonuniformity
	F4.GrayLevelRunLengthMatrix25.333RunPercentage
	F4.GrayLevelRunLengthMatrix25...0RunPercentage
	F4.GrayLevelRunLengthMatrix25..90RunPercentage
	F4.GrayLevelRunLengthMatrix25.333ShortRunHighGrayLevelEmpha
	F4.GrayLevelRunLengthMatrix25...0ShortRunHighGrayLevelEmpha
	F4.GrayLevelRunLengthMatrix25..90ShortRunHighGrayLevelEmpha
**Neighborhood gray-tone difference matrix (NGTDM) 3 and 25**	
1/144	F8.NeighborIntensityDifference25Coarseness

(0.7%)	
**Gradient Orient Histogram**	
32/144 (22.2%)	F1.GradientOrientHistogramKurtosis<br>F1.GradientOrientHistogram35Percentile<br>F1.GradientOrientHistogram40Percentile<br>F1.GradientOrientHistogram45Percentile<br>F1.GradientOrientHistogram50Percentile<br>F1.GradientOrientHistogram55Percentile<br>F1.GradientOrientHistogram60Percentile<br>F1.GradientOrientHistogram65Percentile<br>F1.GradientOrientHistogram70Percentile<br>F1.GradientOrientHistogram75Percentile<br>F1.GradientOrientHistogram80Percentile<br>F1.GradientOrientHistogram85Percentile<br>F1.GradientOrientHistogram90Percentile<br>F1.GradientOrientHistogram95Percentile<br>F1.GradientOrientHistogram30PercentileArea<br>F1.GradientOrientHistogram35PercentileArea<br>F1.GradientOrientHistogram40PercentileArea<br>F1.GradientOrientHistogram45PercentileArea<br>F1.GradientOrientHistogram50PercentileArea<br>F1.GradientOrientHistogram55PercentileArea<br>F1.GradientOrientHistogram60PercentileArea<br>F1.GradientOrientHistogram65PercentileArea<br>F1.GradientOrientHistogram70PercentileArea<br>F1.GradientOrientHistogram75PercentileArea<br>F1.GradientOrientHistogram80PercentileArea<br>F1.GradientOrientHistogram85PercentileArea<br>F1.GradientOrientHistogram90PercentileArea<br>F1.GradientOrientHistogram95PercentileArea<br>F1.GradientOrientHistogram..0.5Quantile<br>F1.GradientOrientHistogram.0.75Quantile<br>F1.GradientOrientHistogram0.975Quantile<br>F1.GradientOrientHistogramSkewness

Supplementary Table S4. Performance metrics with XG Boost models to predict the hot/cold phenotype

Metric	Definition	Min-Max
**accuracy**	Number of correct predictions / total number of input samples = TP+TN/TP+FP+FN+TN	0.35 - 0.75
**precision**	Number of correct positive predictions / number of positive predictions = TP/TP+FP	0.333 - 0.833
**recall**	Number of correct positive predictions / number of all positive samples = TP/TP+FN	0.111 - 1
**F1-score**	Harmonic mean of the precision and the recall = 2*(Recall * Precision) / (Recall + Precision)	0.167 - 0.8
**ROC AUC**	Area under the curve of true positive rate and false positive rate at various thresholds (Range [0,1] if AUC = 1, all predictions are correct)	0.394 - 0.858
**PR AUC**	Area under the curve of precision and recall at various thresholds (Range [0,1])	0.211 - 0.734
